# Supplementary material for: Atomic-resolution mapping of transcription factor-DNA interactions by femtosecond laser crosslinking and mass spectrometry
Source: Nat Commun. 2020 Jun 15;11:3019. doi: 10.1038/s41467-020-16837-x (PMC7295792; doi:10.1038/s41467-020-16837-x)
Supplement: Supplementary file 1 — Supplementary Information File [file 41467_2020_16837_MOESM1_ESM.pdf]

Supplementary Information

Reim et al.: ‘Atomic-resolution mapping of transcription factor-DNA interactions by femtosecond laser crosslinking and mass spectrometry’

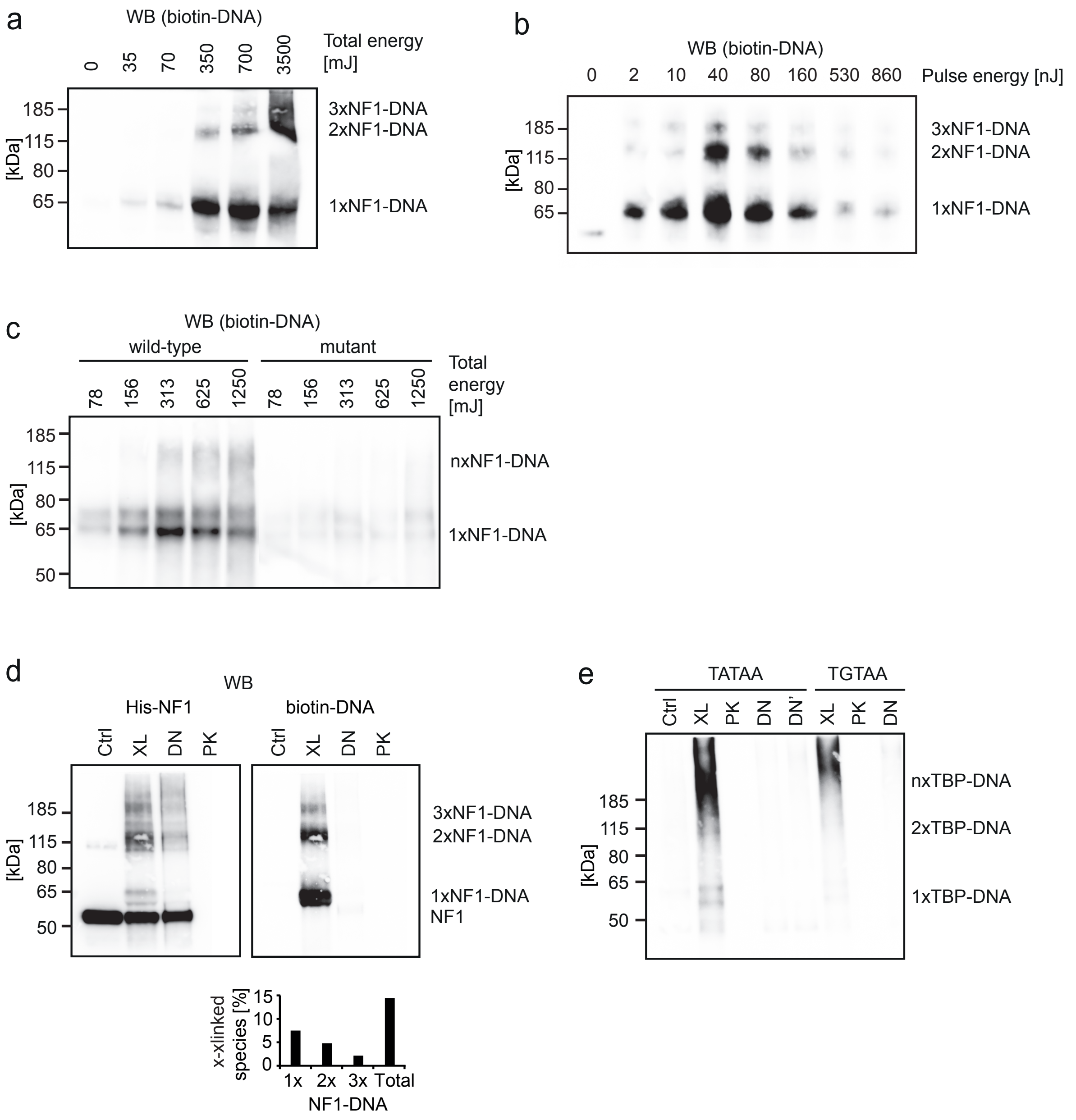

Supplementary Figure 1: Assessment of crosslinking efficiencies. a, Titration of total energy with constant pulse energy of 7 nJ. Full scale version of Western-blot depicted in Fig. 2b. **b,** Titration of pulse energy with constant total energy of 1J. Full scale version of Western-blot depicted in Fig. 2c. **c,** NF1-DNA crosslinking is specific for a wild type consensus motif. Full scale version of Western-blot depicted in Fig. 2d. **d,** Western blot probed by anti-His antibody (left) or Streptavidin-HRP conjugate (right) following membrane stripping. Full scale version of Western-blot depicted in Fig. 2e. Percentage of crosslinked species were extrapolated from the mono-NF1 band reflecting 7.5% of all NF1 (see Fig. 2e) according to their relative intensities in the biotin-DNA blot. **e,** Anti biotin-DNA Western blots of UV-crosslinked TBP-DNA complex with wild type (TATAA) or point-mutated (TGTAA) consensus motif. Full scale version of Western-blot depicted in Fig. 2f.

```
NFIC_PIG      1 MYSSPLCLTQDEFHPFIEALLPHVRAFAYTWFNLQARKRKYFKKHEKRMSKDEERAVKDE
NFIC_HUMAN    1 MYSSPLCLTQDEFHPFIEALLPHVRAFAYTWFNLQARKRKYFKKHEKRMSKDEERAVKDE
NFIC_RAT      1 MYSSPLCLTQDEFHPFIEALLPHVRAFAYTWFNLQARKRKYFKKHEKRMSKDEERAVKDE

NFIC_PIG     61 LLGEKAEVKQKWASRLLAKLRKDIRPECREDFVLAITGKKAPGCVLSNPDQKGKMRRIDC
NFIC_HUMAN   61 LLGEKPEVKQKWASRLLAKLRKDIRPECREDFVLSITGKKAPGCVLSNPDQKGKMRRIDC
NFIC_RAT     61 LLGEKAEVKQKWASRLLAKLRKDIRPECREDFVLAVTGKKAPGCVLSNPDQKGKMRRIDC

NFIC_PIG    121 LRQADKVWRLDLVMVILFKGIPLESTDGERLVKAAQCGHPVLCVQPHHIGVAVKELDLYL
NFIC_HUMAN  121 LRQADKVWRLDLVMVILFKGIPLESTDGERLVKAAQCGHPVLCVQPHHIGVAVKELDLYL
NFIC_RAT    121 LRQADKVWRLDLVMVILFKGIPLESTDGERLVKAAACAHPVLCVQPHHIGVAVKELDLYL

NFIC_PIG    181 AYFVRERDAEQSGSPRAGMGSDQEDSKPITLDTTDFQESFVTSGVFSVTELIQVSRTPVV
NFIC_HUMAN  181 AYFVRERDAEQSGSPRTGMGSDQEDSKPITLDTTDFQESFVTSGVFSVTELIQVSRTPVV
NFIC_RAT    181 AYFVRERDAEQSSSPRTGVGSDQEDSKPITLDTTDFQESFVTSGVFSVTELIQVSRTPVV

NFIC_PIG    241 TGTGPNFSLGELQGHMAYDLNPASTGMRRTLPSTSSSGSKRHKSGSMEEDVDTSPGGDYY
NFIC_HUMAN  241 TGTGPNFSLGELQGHLAYDLNPASTGLRRTLPSTSSSGSKRHKSGSMEEDVDTSPGGDYY
NFIC_RAT    241 TGTGPNFSLGELQGHLAYDLNPASAGMRRTLPSTSSSGSKRHKSGSMEEDVDTSPGGDYY

NFIC_PIG    301 TSPSSPTSSNRNWTEDMEGGISSPVKKTEMDKSPFNSPSPQDSPRLSSFTQHHRPVIAVH
NFIC_HUMAN  301 TSPSSPTSSSRNWTEDMEGGISSPVKKTEMDKSPFNSPSPQDSPRLSSFTQHHRPVIAVH
NFIC_RAT    301 TSPNSPTSSSRNWTEDMEGGISSPVKKTEMDKSPFNSPSPQDSPRLSSFTQHHRPVIAVH

NFIC_PIG    361 SGIARSPHPSSALHFPTTSILPQTASTYFPHTAIRYPPHLNPQDPLKDLVSLACDPASQQ
NFIC_HUMAN  361 SGIARSPHPSSALHFPTTSILPQTASTYFPHTAIRYPPHLNPQDPLKDLVSLACDPASQQ
NFIC_RAT    361 SGIARSPHPSSALHFPATPILPQTASTYFPHTAIRYPPHLNPQDPLKDLVSLACDPATQQ

NFIC_PIG    421 PGPLNGSGQLKMSSHCLSAQMLAPPPPGLPRLALPPATKPT--SEGGSSSPTSPSYSTPG
NFIC_HUMAN  421 PGPLNGSGQLKMPSHCLSAQMLAPPPPGLPRLALPPATKPATTSEGGATSPTSPSYSPPD
NFIC_RAT    421 PGPPA-----------------------L-RPARPLQTVPL-------------------

NFIC_PIG    479 TSPANRSFVGLGPRDPTGIYQAQSWYLG
NFIC_HUMAN  481 TSPANRSFVGLGPRDPAGIYQAQSWYLG
NFIC_RAT    438 ------------------------WD--
```

Supplementary Figure 2: Sequence alignment of Nuclear Factor 1/C in pig, human and rat. Crosslinked peptides of the pig Nuclear Factor 1/C are highlighted in pale blue. The crosslinked amino acid is indicated by a red star above the amino acid or above the bracket around a stretch of amino acids. Amino acids are depicted in red if their mutation was found to significantly reduce DNA binding in rat NF1[1].

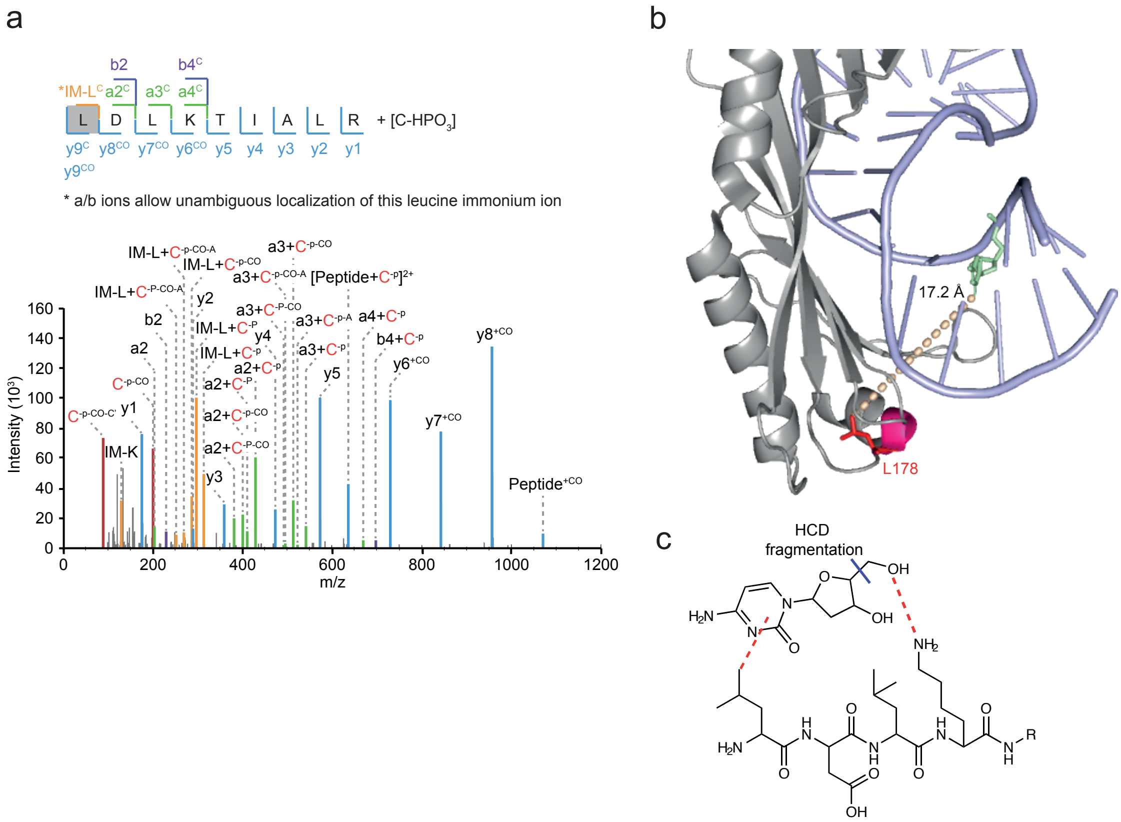

Supplementary Figure 3: Spectrum and structural information of the crosslinked LDLKTIALR peptide. a, MS2 ion series and spectra of the peptide LDLKTIALR crosslinked to a cytosine. Abbreviations in the MS2 spectra and MS2 ion series: +CO: adduct of carbon monoxide. Other abbreviations as in Fig. 4 and 5. **b,** Location of the crosslink of the amino acid L178 to deoxycytidine (light green) in the crystal structure (PDB ID: 1C9B[2]). The crosslinked amino acid is depicted in red and the peptide 178LDLKTIALR186 in pink. **c,** Proposed scheme of the double crosslink of L178 and K181 to the deoxycytidine and creation of the CO adduct on the y6-y9 ion series after HCD fragmentation as observed in Supplementary Figure 3a. The blue line indicates bond breakage after HCD fragmentation. Dashed, red lines represent the possible covalent bonds of the two amino acids to the nucleotide. R: amino acids TIALR.

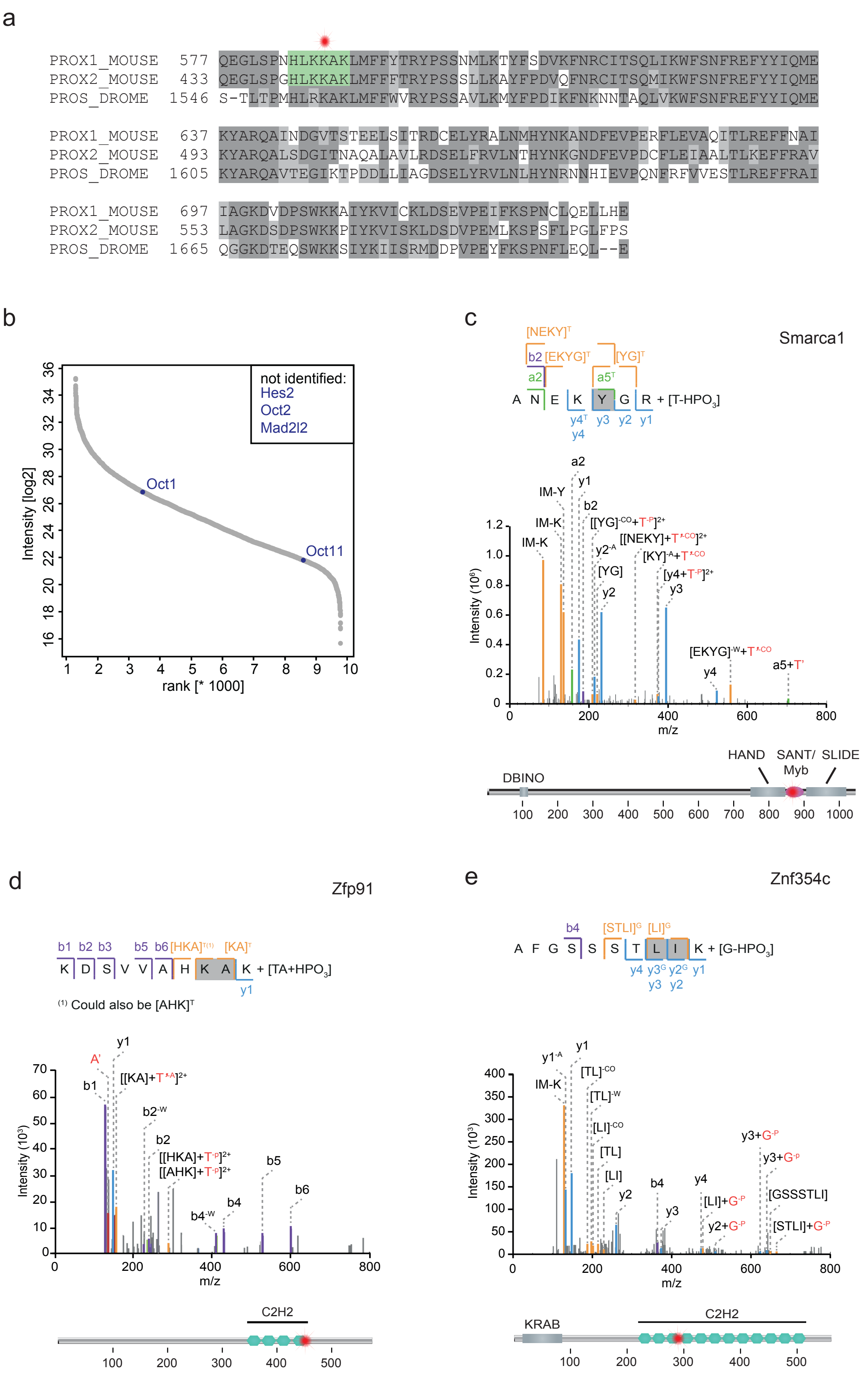

Supplementary Figure 4: Additional data for in-vivo fliX-MS in mouse embryonic stem cells. a, Sequence alignment of mouse Prox1 and Prox2 and *D. melanogaster* Prospero. The identified

peptide and the crosslinked amino acids are highlighted in green or with a red star, respectively. **b,** Ranked log2 protein intensity of a deep proteomic analysis of mouse ES cells (E14TG2a) identifies expression of Oct1 and Oct11. **c-e,** MS/MS spectra of high-confidence crosslinks obtained from the in-vivo crosslinking experiment including the global transcription factor Smarca1 and the two zinc finger proteins Zfp91 and Znf354c. Abbreviations in the MS/MS spectra as in Fig. 4 and schematic representation of the crosslink location on the protein as in Fig. 6d-e.

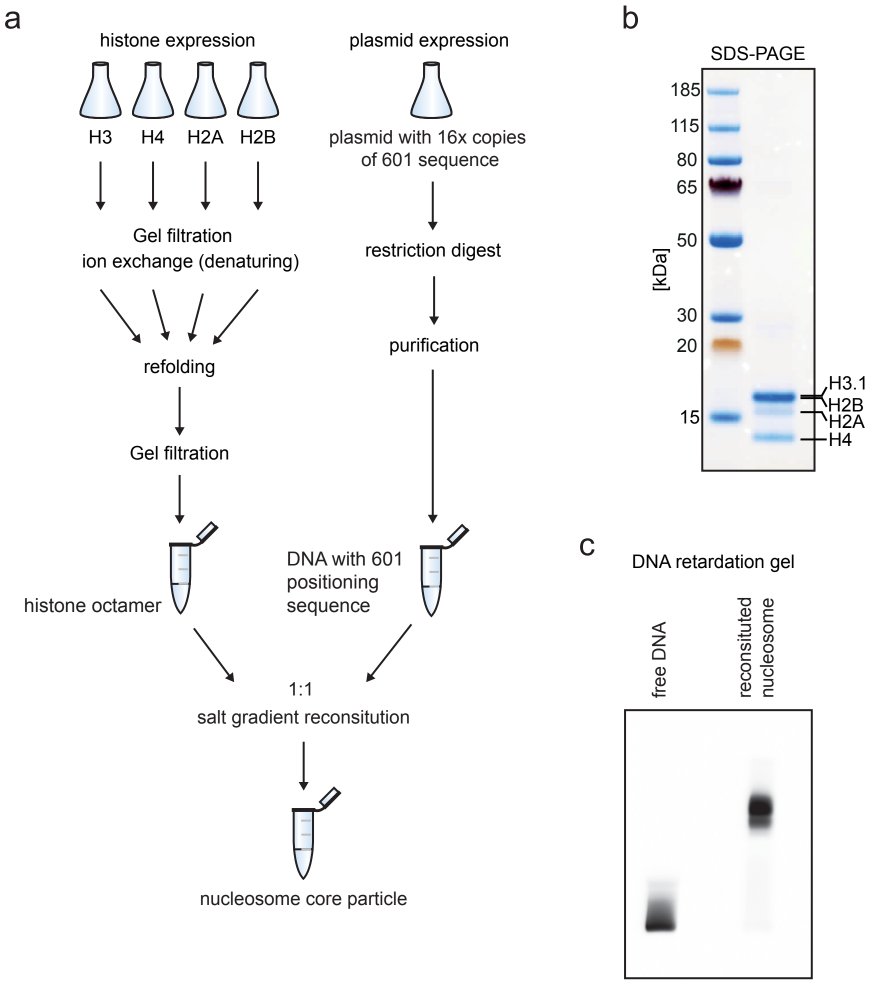

Supplementary Figure 5: Reconstitution of recombinant human nucleosome core particles. a, Schematic workflow of experimental workflow to generate recombinant core particles adapted from [3-5]. Individual histone proteins are expressed in E. coli and sequentially purified by Gel filtration and Strong Cation Exchange chromatography. Histones are combined and refolded followed by purification of histone octamers by Gel filtration. The DNA is generated as plasmid containing 16 copies of the strong nucleosome positioning 601 sequence. After restriction digest, individual fragments were purified, mixed together with the histone octamer in a 1:1 molar ratio, and nucleosome core particles reconstituted by dialysis applying a salt gradient. **b,** Reconstituted nucleosomes were separated on a SDS-PAGE and proteins visualized by Colloidal Blue Staining. **c,** Comparison of reconstituted nucleosome or the same amount of 601 DNA on a DNA retardation gel revealed 100% reconstitution efficiency.

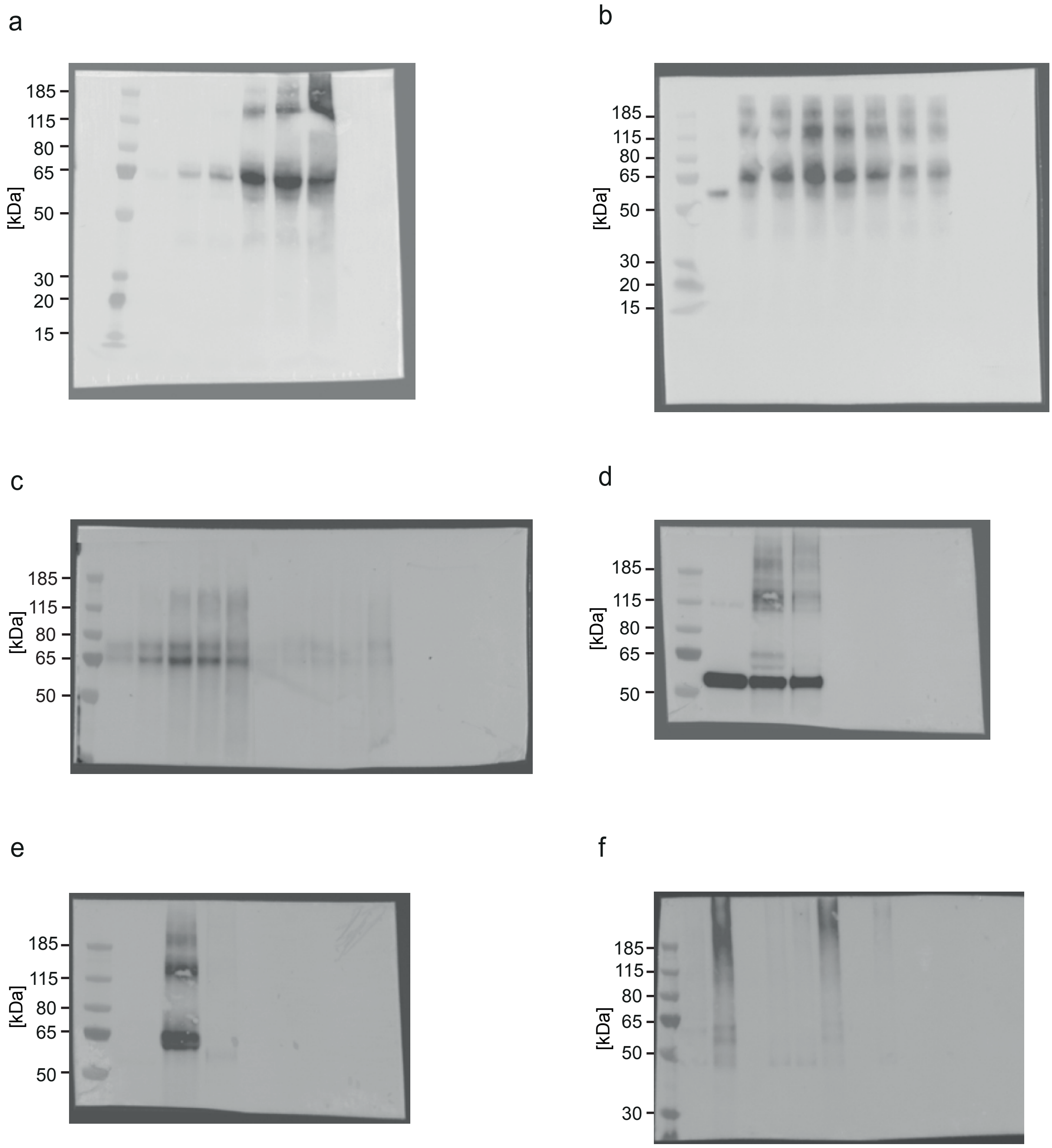

Supplementary Figure 6: Original Western blots. a, Full scale, original version of Western-blot shown in Figure 2b and Supplementary Figure 1a. **b**, Full scale, original version of Western-blot depicted in Figure 2c and Supplementary Figure 1b. **c**, Full scale, original version of Western-blot shown in Figure 2d and Supplementary Figure 1c. **d**, Full scale, original version of anti His-NF1 Western-blot shown in Figure 2e (left blot) and Supplementary Figure 1d (left blot). **e**, Full scale, original version of anti biotin-DNA Western-blot shown in Figure 2e (right blot) and Supplementary Figure 1d (right blot). **f**, Full scale, original version of western blot probed by Streptavidin-HRP conjugate depicted in Figure 2f and Supplementary Figure 1e.

Supplementary References

1. Dekker, J., van Oosterhout, J.A. & van der Vliet, P.C. Two regions within the DNA binding domain of nuclear factor I interact with DNA and stimulate adenovirus DNA replication independently. *Mol Cell Biol* **16**, 4073-80 (1996).
2. Tsai, F.T.F. & Sigler, P.B. Structural basis of preinitiation complex assembly on human Pol II promoters. *Embo Journal* **19**, 25-36 (2000).
3. Luger, K., Rechsteiner, T.J. & Richmond, T.J. Expression and Purification of Recombinant Histones and Nucleosome Reconstitution. in *Chromatin Protocols* (ed. Becker, P.B.) 1-16 (Humana Press, Totowa, NJ, 1999).
4. Dyer, P.N. et al. Reconstitution of nucleosome core particles from recombinant histones and DNA. *Methods Enzymol* **375**, 23-44 (2004).
5. Bartke, T. et al. Nucleosome-interacting proteins regulated by DNA and histone methylation. *Cell* **143**, 470-84 (2010).
