# Supplementary material for: Atomic-resolution mapping of transcription factor-DNA interactions by femtosecond laser crosslinking and mass spectrometry
Source: Nat Commun. 2020 Jun 15;11:3019. doi: 10.1038/s41467-020-16837-x (PMC7295792; doi:10.1038/s41467-020-16837-x)
Supplement: Supplementary file 3 — Description of Additional Supplementary Files [file 41467_2020_16837_MOESM3_ESM.docx]

**Description of Additional Supplementary Files**

File name: Supplementary Data1.pdf

Description:

**Supplementary Data 1: Annotated MS/MS spectra of all crosslinks from this study.** All annotated peaks are labeled with the corresponding fragment ion together with the observed experimental mass. The following abbreviations were used: A: Deoxyadenosine monophosphate, C: Deoxycytidine monophosphate, G: Deoxyguanosine monophosphate, T: Thymidine monophosphate, X’: Nucleobase of either of the Nucleotides (X = A, C, G or T), -A: Neutral loss of NH_3_, -Ca: Neutral loss of isocyanic acid from Carbamyl group, -CO: Neutral loss of Carbon monoxide, +CO: Adduct with Carbon monoxide, -HP: Neutral loss of hydrogen peroxide on trioxidized cysteins, -p: Neutral loss of HPO_3_, -P: Neutral loss of H_3_PO_4_, Ox: Oxidation of methionine, Triox/Tox: Trioxidation of cysteine, -W: Neutral loss of H_2_O, +W: Adduct with H_2_O, Asterisk: Neutral loss of H_2_SO_3_ on trioxidized cysteine, Caret: Neutral loss of CH_4_SO of oxidized methionine.

File name: Supplementary Data2.xlsx

Description:

**Supplementary Data 2: Full annotation of theoretical and observed fragment ions of all DNA crosslinked peptides.** The same abbreviations apply as in Supplementary Data 1.
