# Supplementary material for: Atomic-resolution mapping of transcription factor-DNA interactions by femtosecond laser crosslinking and mass spectrometry
Source: Nat Commun. 2020 Jun 15;11:3019. doi: 10.1038/s41467-020-16837-x (PMC7295792; doi:10.1038/s41467-020-16837-x)

Supplementary Data 1

Reim et al.: 'Atomic-resolution mapping of transcription factor-DNA interactions by femtosecond laser crosslinking and mass spectrometry'

Supplementary Data 1: Annotated MS/MS spectra of all crosslinks from this study. All annotated peaks are labeled with the corresponding fragment ion together with the observed experimental mass. The following abbreviations were used: A: Deoxyadenosine monophosphate, C: Deoxycytidine monophosphate, G: Deoxyguanosine monophosphate, T: Thymidine monophosphate, X': Nucleobase of either of the Nucleotides (X = A, C, G or T), -A: Neutral loss of NH_3, -Ca: Neutral loss of isocyanic acid from Carbamyl group, -CO: Neutral loss of Carbon monoxide, +CO: Adduct with Carbon monoxide, -HP: Neutral loss of hydrogen peroxide on trioxidized cysteins, -p: Neutral loss of HPO_3, -P: Neutral loss of H_3PO_4, Ox: Oxidation of methionine, Triox/Tox: Trioxidation of cysteine, -W: Neutral loss of H_2O, +W: Adduct with H_2O, Asterisk: Neutral loss of H_2SO_3 on trioxidized cysteine, Caret: Neutral loss of CH_4SO of oxidized methionine.

DCP #1

[21]KVLRDNIQGITK^{Carb}PAIR[36] + [ACCG-H_3PO_4]

Histone H4 (P62805)

m(calculated)			Δm [ppm]	experimental	
peptide	DNA	cross-link		m/z	charge
1864.1007	1140.2365	3004.3372	6.2	1002.4468	3

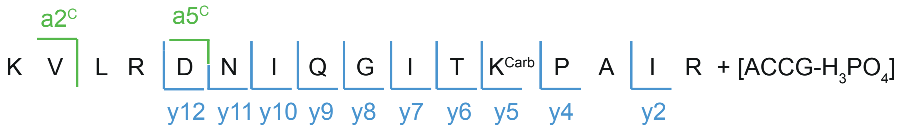

C observed as cytosine crosslink

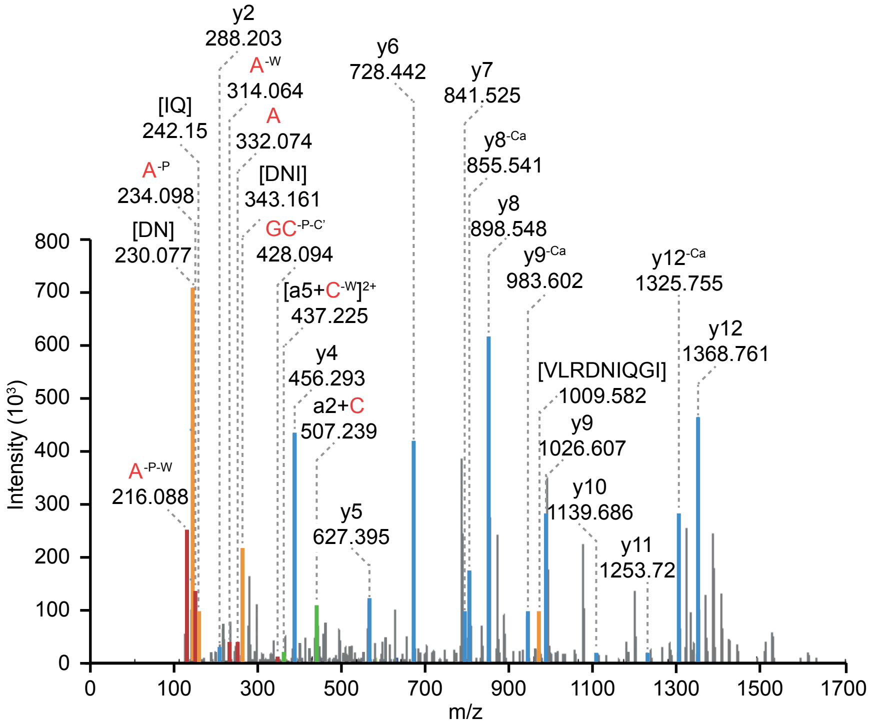

DCP #2

^{22}VLRDNIQGITK^{Carb}PAIR^{36} + [CT-H_2O]

Histone H4 (P62805)

m(calculated)			Δm [ppm]	experimental	
peptide	DNA	cross-link		m/z	charge
1736.0057	593.0924	2329.0981	9.5	777.3659	3

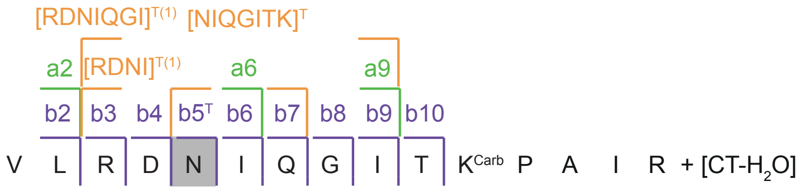

^{T} observed as thymine crosslink, $^{(1)}$ Could also be [LRDN]^{T}/[LRDNIQG]^{T}

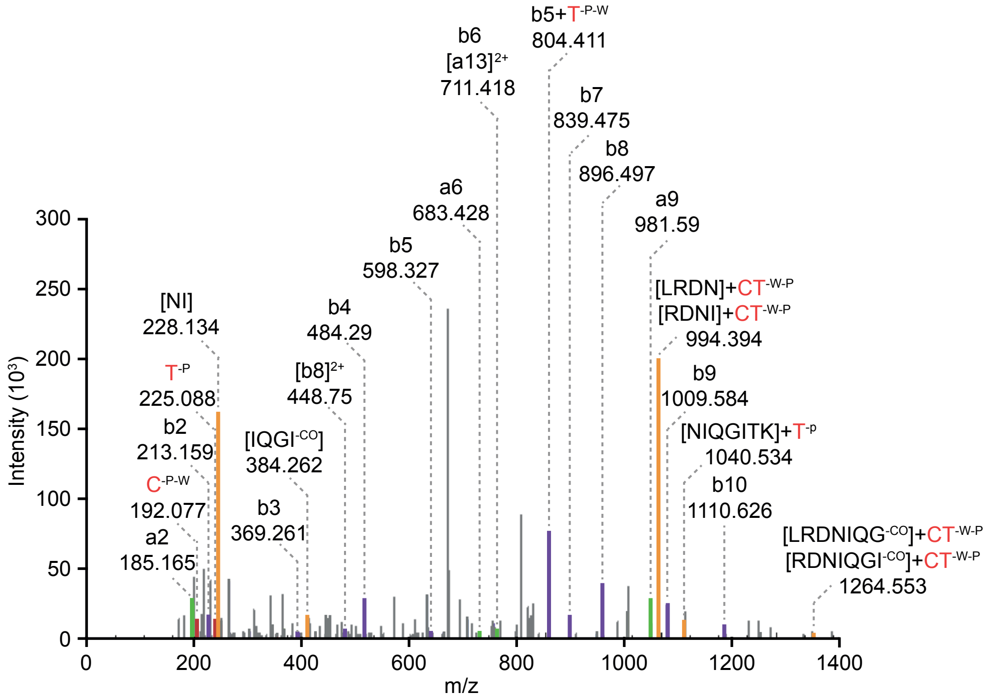

DCP #3

[29]SAPATGGVK[37] + [C-HPO_3]

Histone H3.1 (P68431)

m(calculated)			Δm [ppm]	experimental	
peptide	DNA	cross-link		m/z	charge
786.4236	227.0906	1013.5142	1.6	507.7652	2

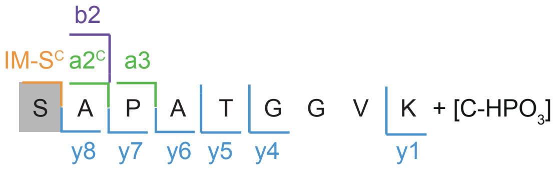

[C] observed as cytosine crosslink

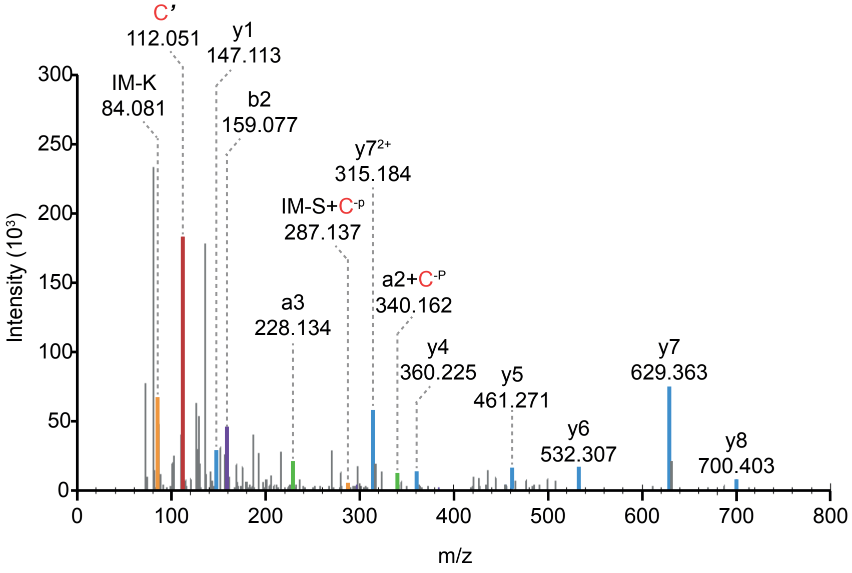

DCP #4

^{74}EIAQDFK^{80} + [AT-HPO$_3$]

Histone H3.1 (P68431)

m(calculated)			Δm [ppm]	experimental	
peptide	DNA	cross-link		m/z	charge
849.4232	555.1479	1404.5711	6.5	703.2883	2

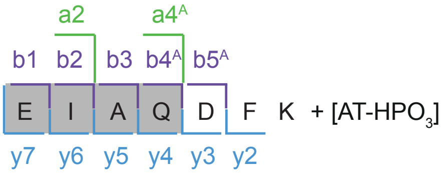

^{A} observed as adenine crosslink

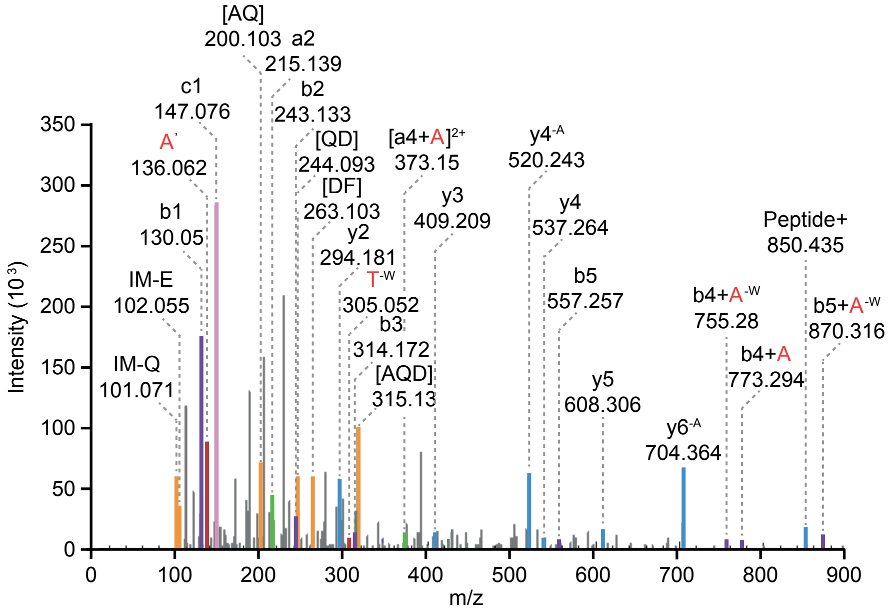

DCP #5

[118]VTIM[Ox]PKDIQLAR[129] + [C-HPO_3]

Histone H3.1 (P68431)

m(calculated)			Δm [ppm]	experimental	
peptide	DNA	cross-link		m/z	charge
1399.7857	227.0906	1626.8763	6.7	543.3030	3

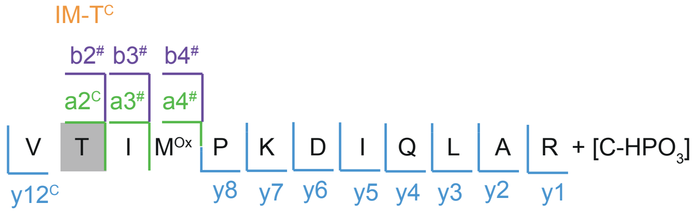

^C observed as cytosine crosslink

^# observed with mass shift of 184.095

^ neutral loss of CH_4SO on oxidized methionine

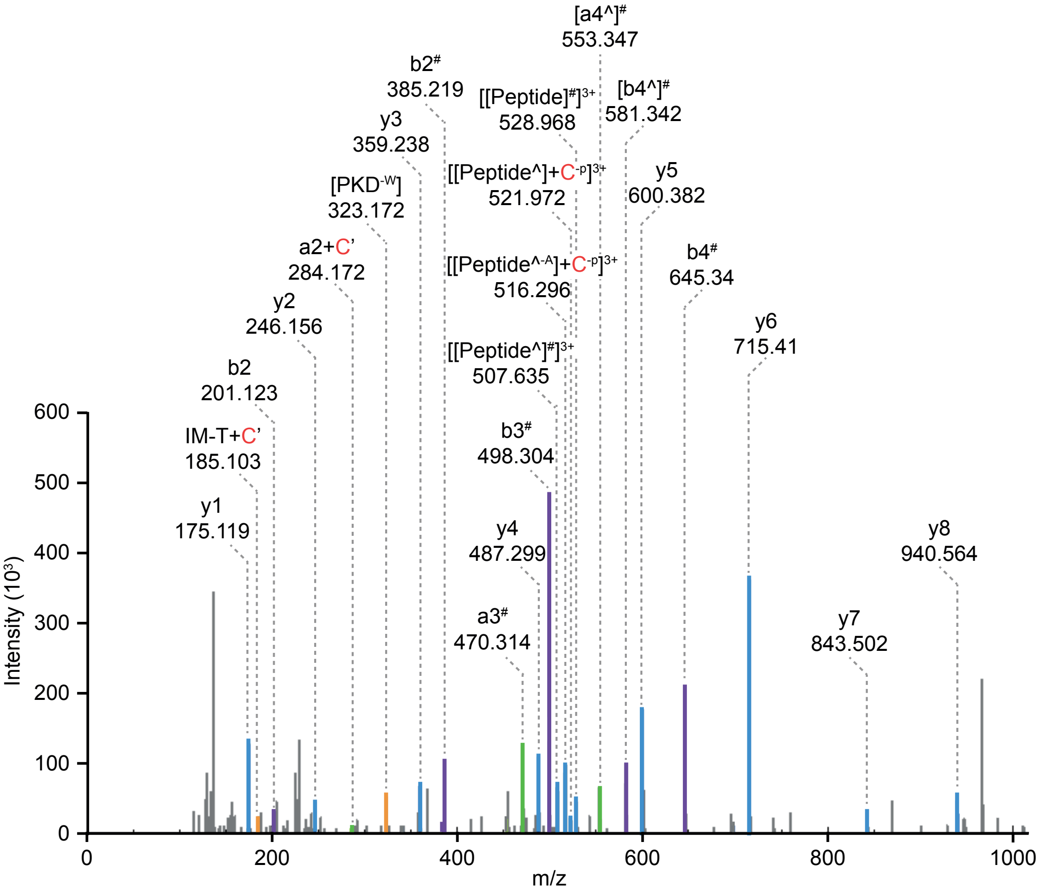

DCP #6

^{45}VLKQVHPDTGISSK^{58} + [AAGT+HPO$_3$]

Histone H2B-1K (O60814)

m(calculated)			Δm [ppm]	experimental	
peptide	DNA	cross-link		m/z	charge
1507.8358	1357.1907	2865.0265	4.6	1433.5139	2

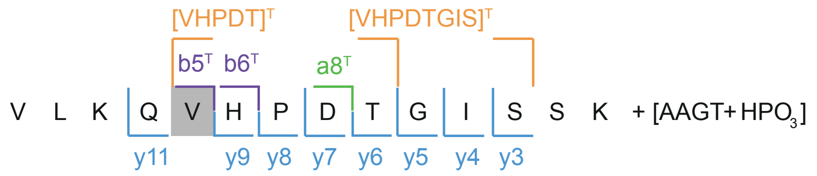

T observed as thymine crosslink

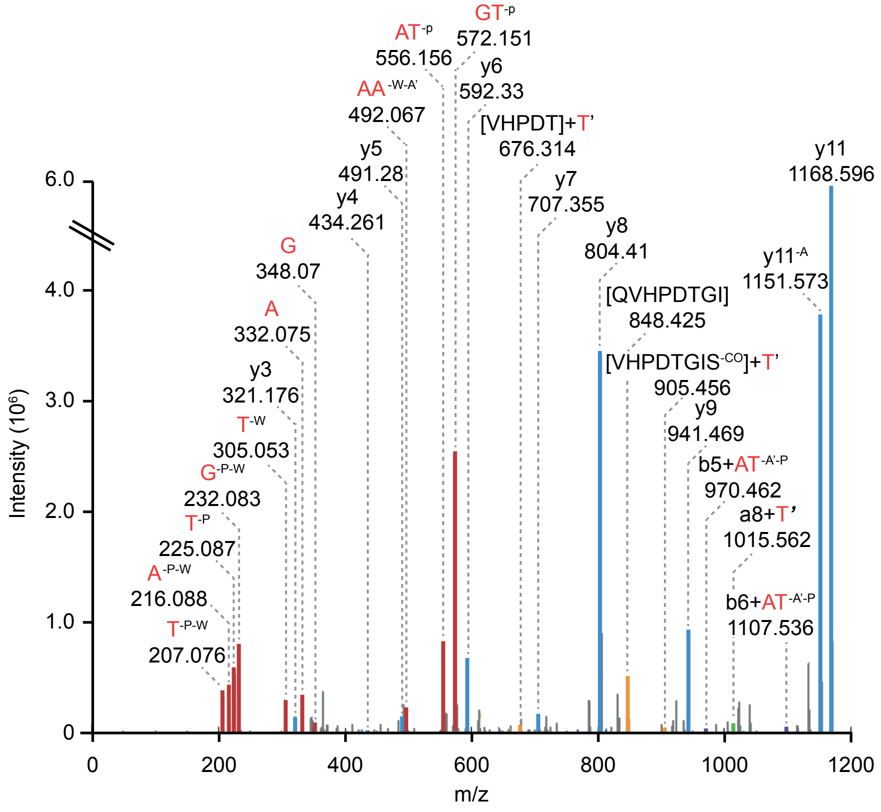

DCP #7

^{45}VLKQVHPDTGISSK^{58} + [AGGT+HPO_3]

Histone H2B-1K (O60814)

m(calculated)			Δm [ppm]	experimental	
peptide	DNA	cross-link		m/z	charge
1507.8358	1373.1856	2881.0214	1.7	1441.5155	2

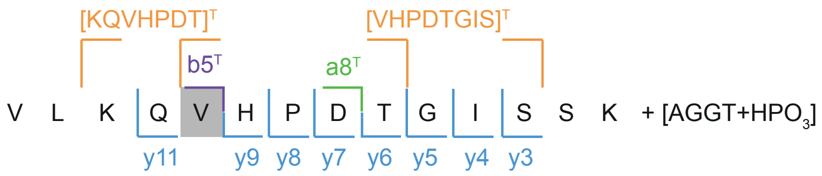

T observed as thymine crosslink

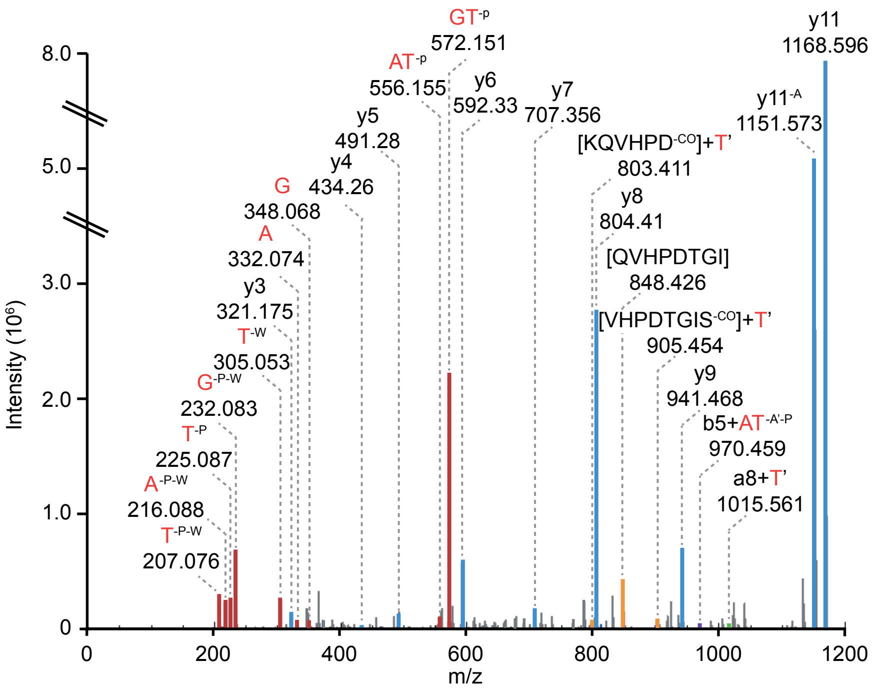

DCP #8

[110]HAVSEGTK[Carb]AVTK[121] + [GT]

Histone H2B (O60814)

m(calculated)			Δm [ppm]	experimental	
peptide	DNA	cross-link		m/z	charge
1269.6677	651.1091	1920.7768	1.7	961.3941	2

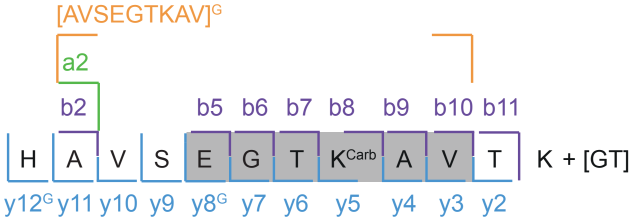

G observed as guanine crosslink

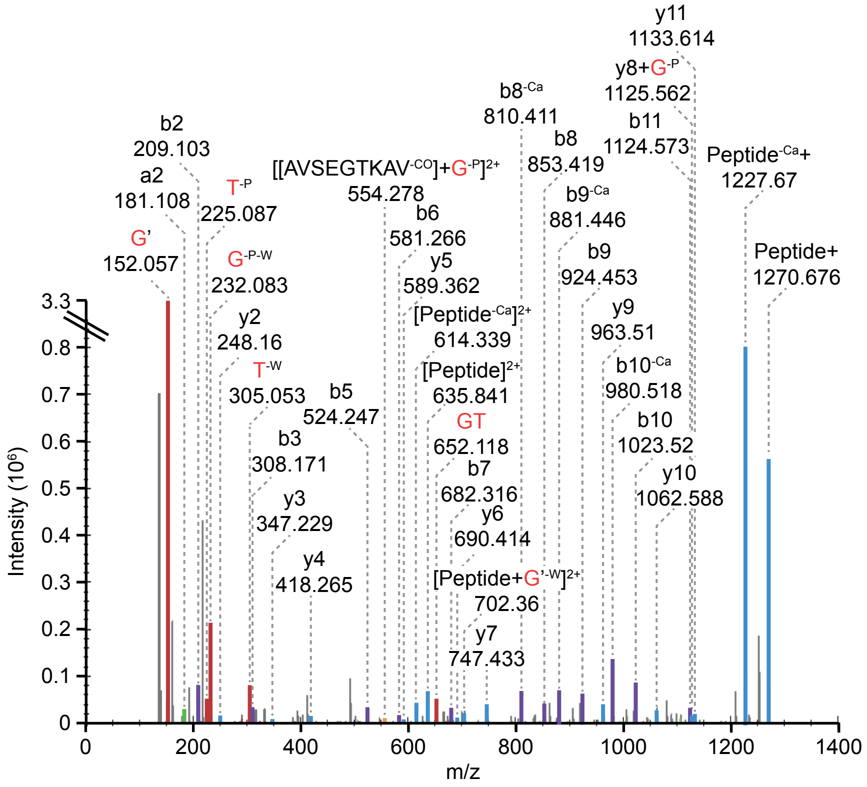

DCP #9

^{79}IIPRHLQLAIRNDEELNK^{96} + [AT]

Histone H2A (P04908)

m(calculated)			Δm [ppm]	experimental	
peptide	DNA	cross-link		m/z	charge
2171.2175	635.1142	2806.3317	8.3	702.5960	4

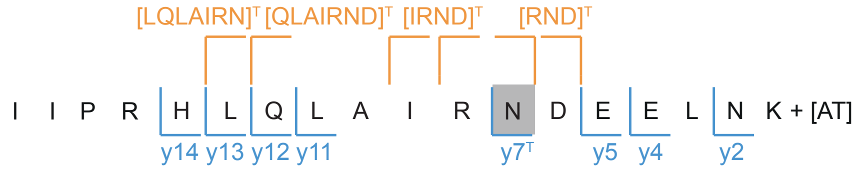

T observed as thymine crosslink

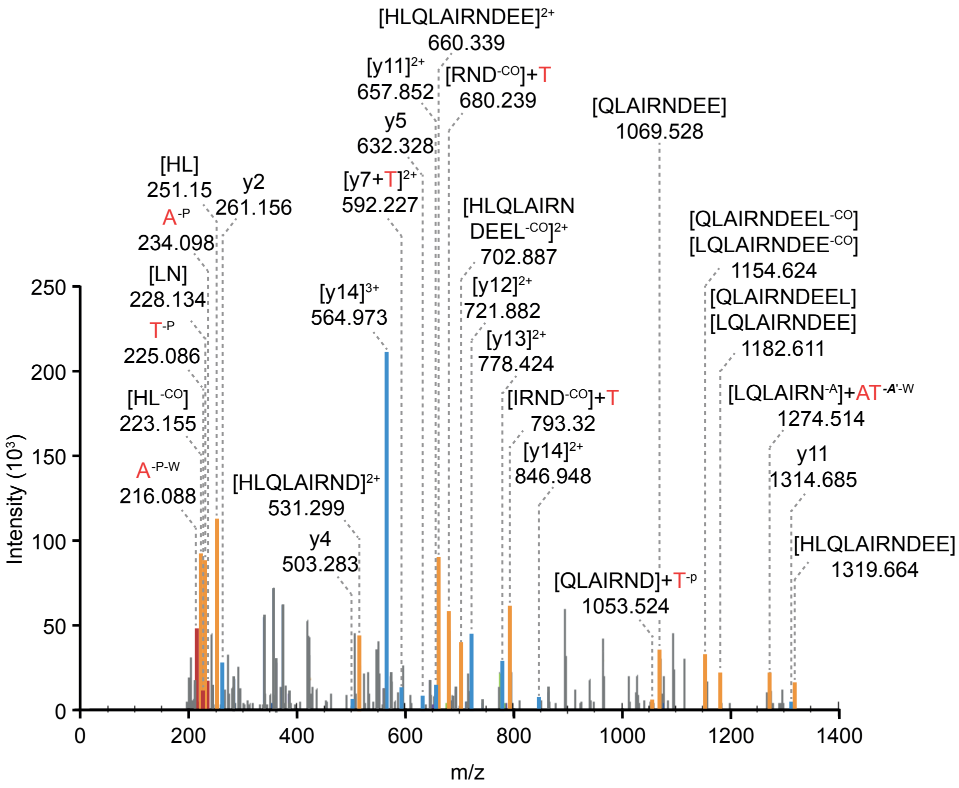

DCP #10

[81]HLQLAIRNDEELNK[Carb,96] + [T-HPO$_3$]

Histone H2A (P04908)

m(calculated)			Δm [ppm]	experimental	
peptide	DNA	cross-link		m/z	charge
1734.9013	242.0903	1976.9916	10.0	660.0110	3

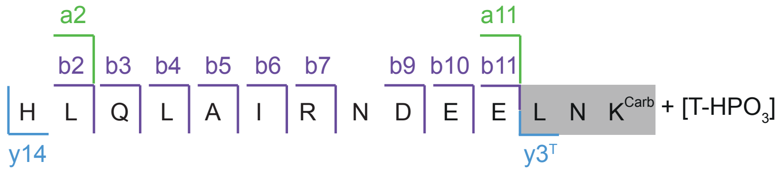

[T] observed as thymine crosslink

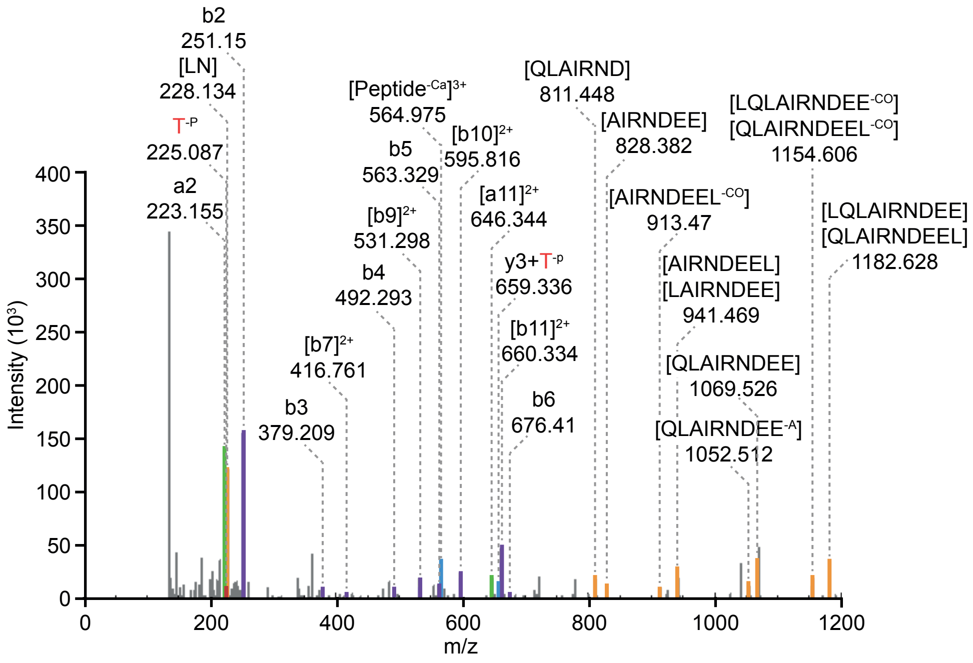

DCP #11

^{81}HLQLAIR^{89} + [AAT-HPO_3]

Histone H2A (P04908)

m(calculated)			Δm [ppm]	experimental	
peptide	DNA	cross-link		m/z	charge
849.5185	868.2055	1717.724	2.6	573.5804	3

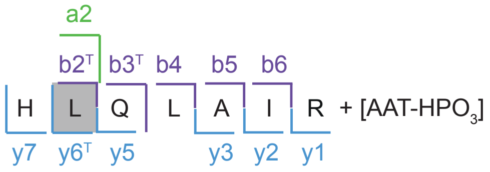

^{T} observed as thymine crosslink

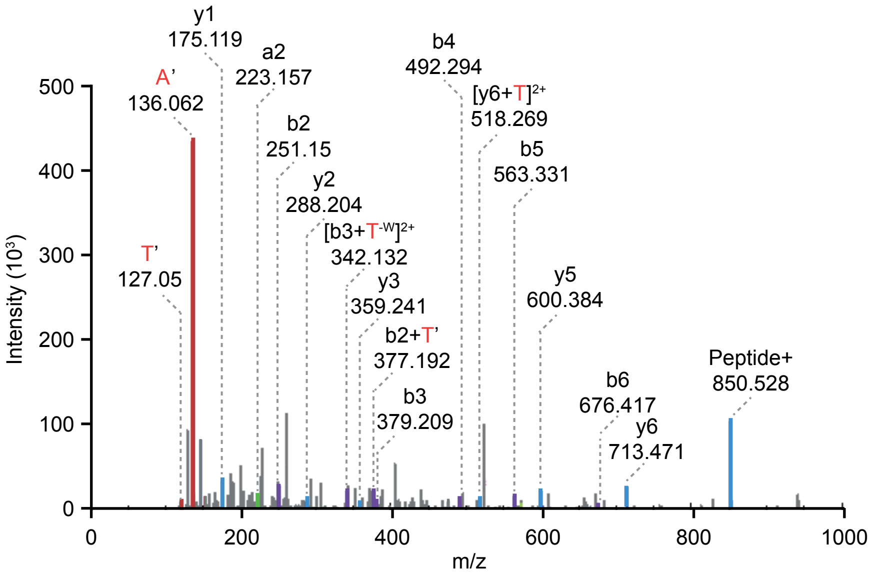

DCP #12

^{90}NDEELNK^{96} + [AC]

Histone H2A (P04908)

m(calculated)			Δm [ppm]	experimental	
peptide	DNA	cross-link		m/z	charge
860.3876	620.1145	1480.5021	1.1	741.2575	2

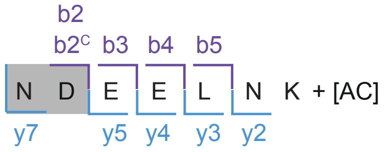

C observed as cytosine crosslink

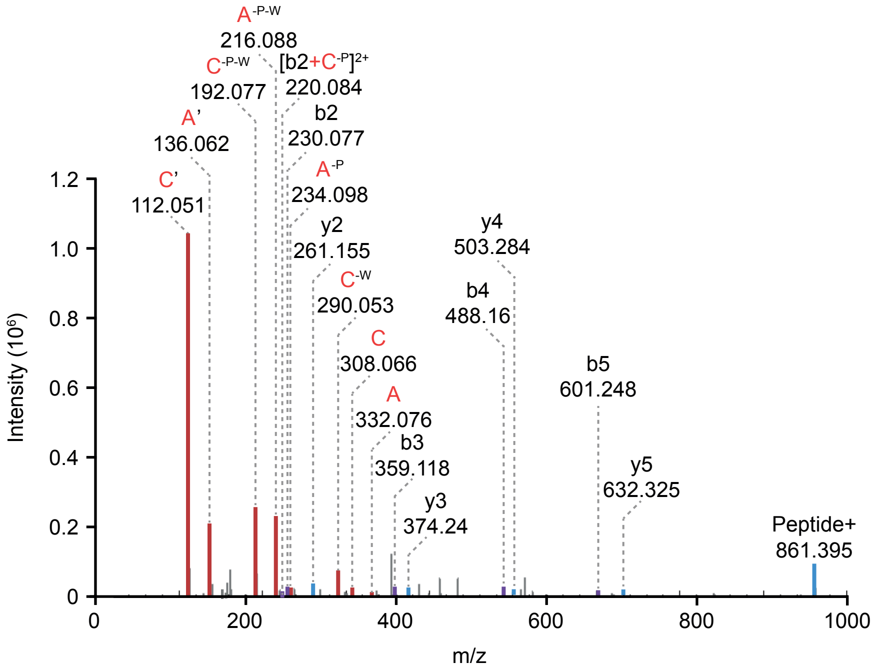

DCP #13

^{83}DIRPECR^{89} + [CCT]

Nuclear Factor 1C (P21999)

m(calculated)			Δm [ppm]	experimental	
peptide	DNA	cross-link		m/z	charge
887.4283	900.1494	1787.5777	6.0	894.7907	2

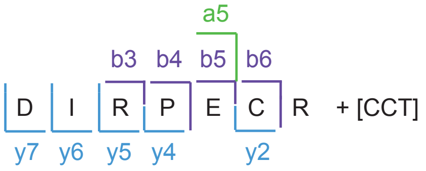

^{C} observed as cytosine crosslink

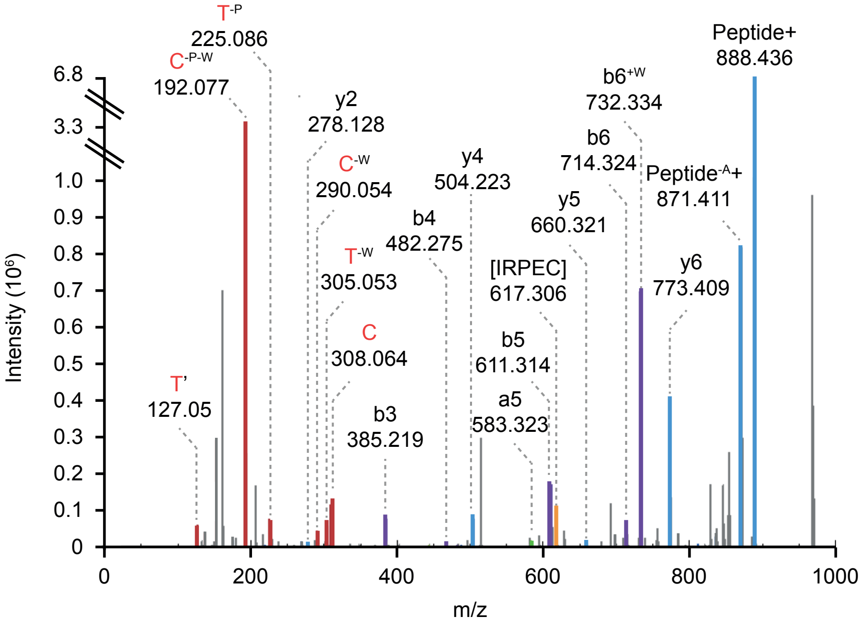

DCP #14

^{101}APGC^{Triox}VLSNPDQK^{112} + [C]

Nuclear Factor 1C (P21999)

m(calculated)			Δm [ppm]	experimental	
peptide	DNA	cross-link		m/z	charge
1275.5765	307.0569	1582.6335	6.4	792.3290	2

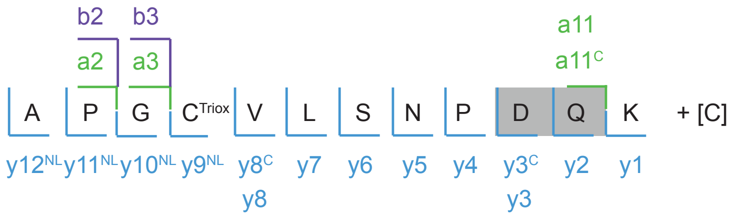

^{C} observed as cytosine crosslink

^{NL} observed after Neutral Loss of H_2SO_3(*) or H_2O_2(HP)

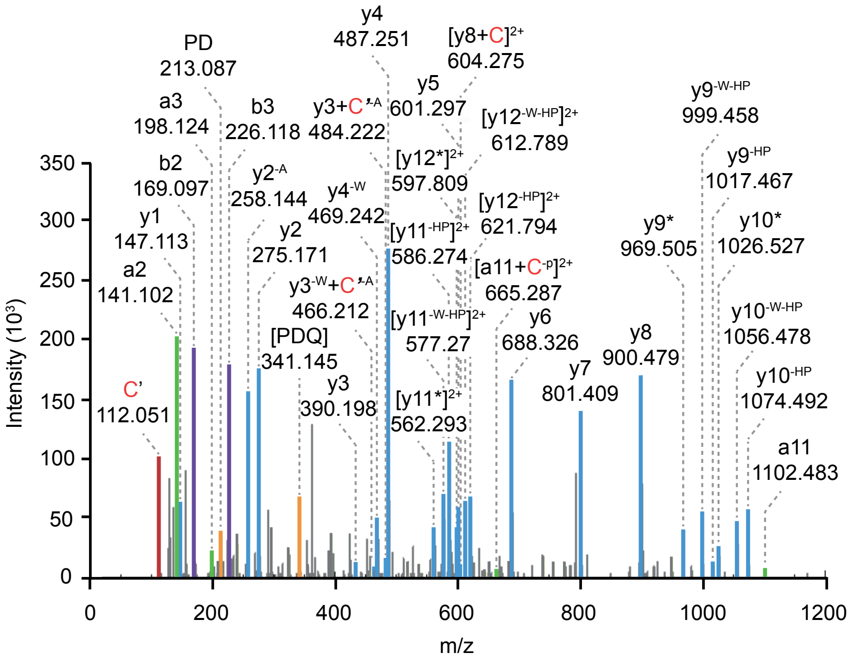

DCP #15

[117]RIDCLR[122] + [TT]

Nuclear Factor 1C (P21999)

m(calculated)			Δm [ppm]	experimental	
peptide	DNA	cross-link		m/z	charge
774.417	626.1026	1400.5197	8.2	701.2614	2

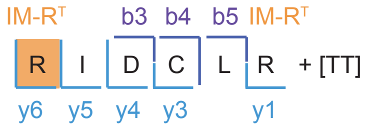

IM-R^T: observed as thymine crosslink on arginine immonium ion

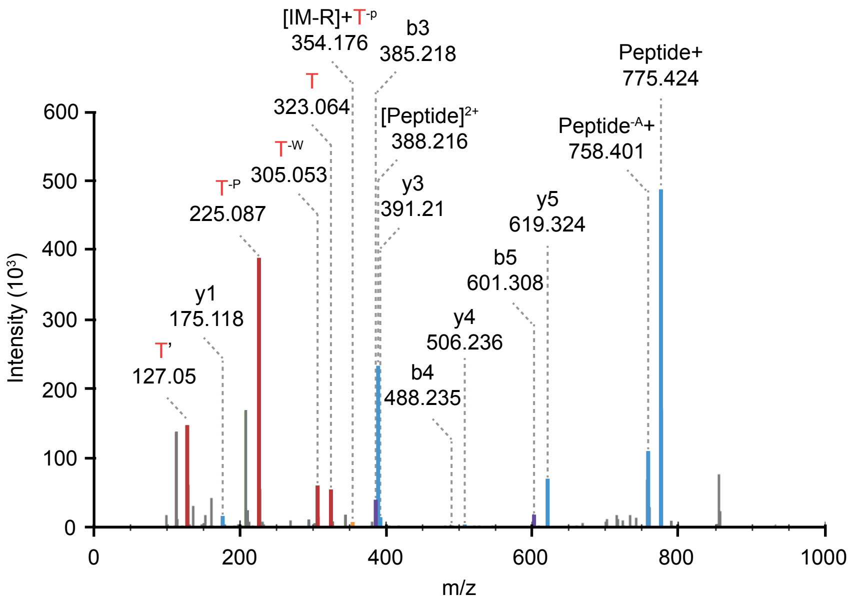

DCP #16

^{140}GIPLESTDGER^{150} + [AC-H_3PO_4]

Nuclear Factor 1C (P21999)

m(calculated)			Δm [ppm]	experimental	
peptide	DNA	cross-link		m/z	charge
1172.5673	522.1376	1694.705	6.3	848.3651	2

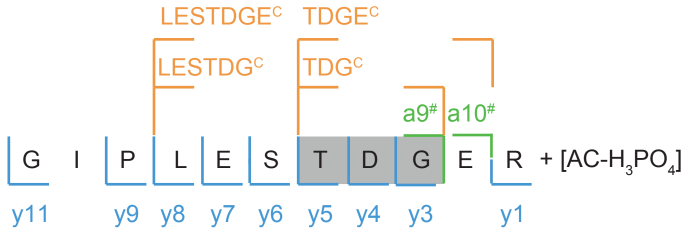

^{C} observed as cytosine crosslink

$^{\#}$ neutral loss of 98.005

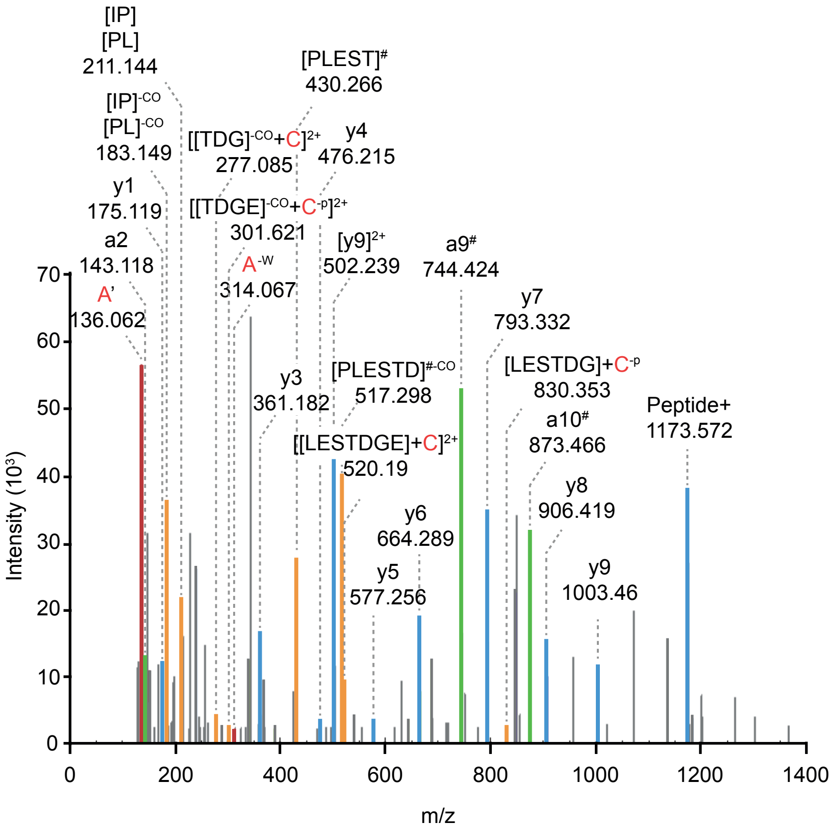

DCP #17

^{154}AAQCGHPVLCVQPHHIGVAVK^{174} + [GT-HPO$_3$]

Nuclear Factor 1C (P21999)

m(calculated)			Δm [ppm]	experimental	
peptide	DNA	cross-link		m/z	charge
2211.1041	571.1428	2782.2469	5.5	928.4280	3

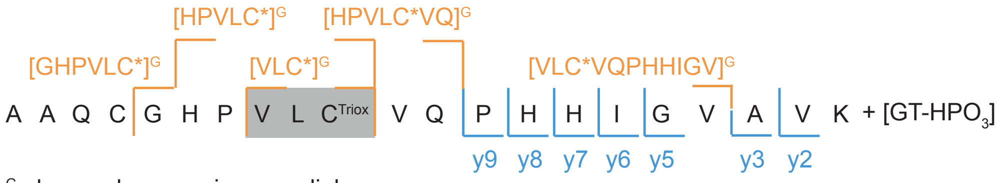

^{G} observed as guanine crosslink

* loss of H_2SO_3 on trioxidized cysteine

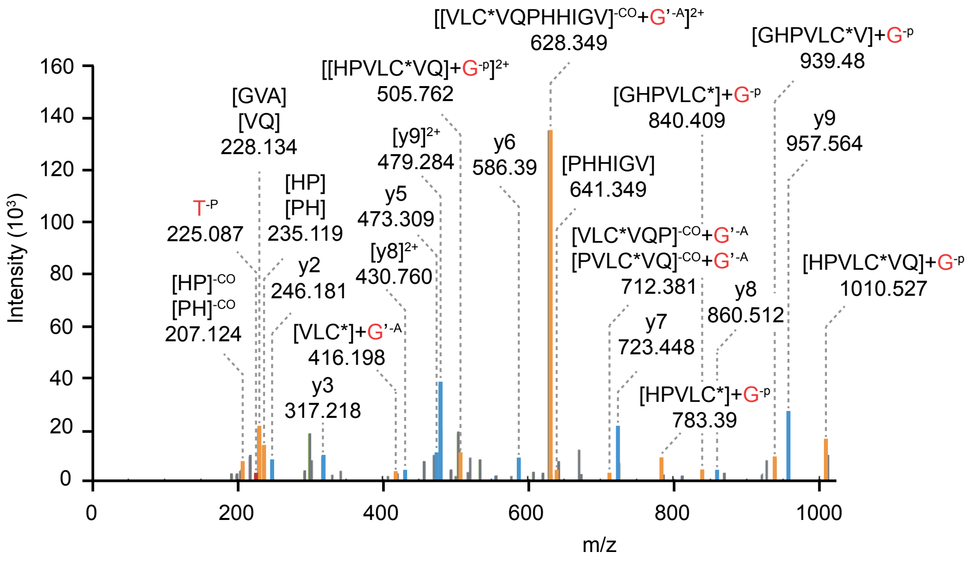

DCP #18

^{209}TTALIFSSGKM^{Ox}VC^{Triox}TGAK^{225} + [AC-HPO_3]

TATA-box-binding protein (P20226)

m(calculated)			Δm [ppm]	experimental	
peptide	DNA	cross-link		m/z	charge
1777.859	540.1482	2318.0072	8.5	773.6829	3

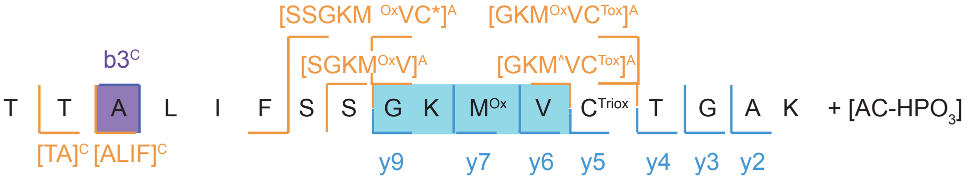

^{C} observed as cytosine crosslink

^{A} observed as adenine crosslink

* neutral loss of H_2SO_3 on trioxidized cysteine

^ neutral loss of CH_4SO on oxidized methionine

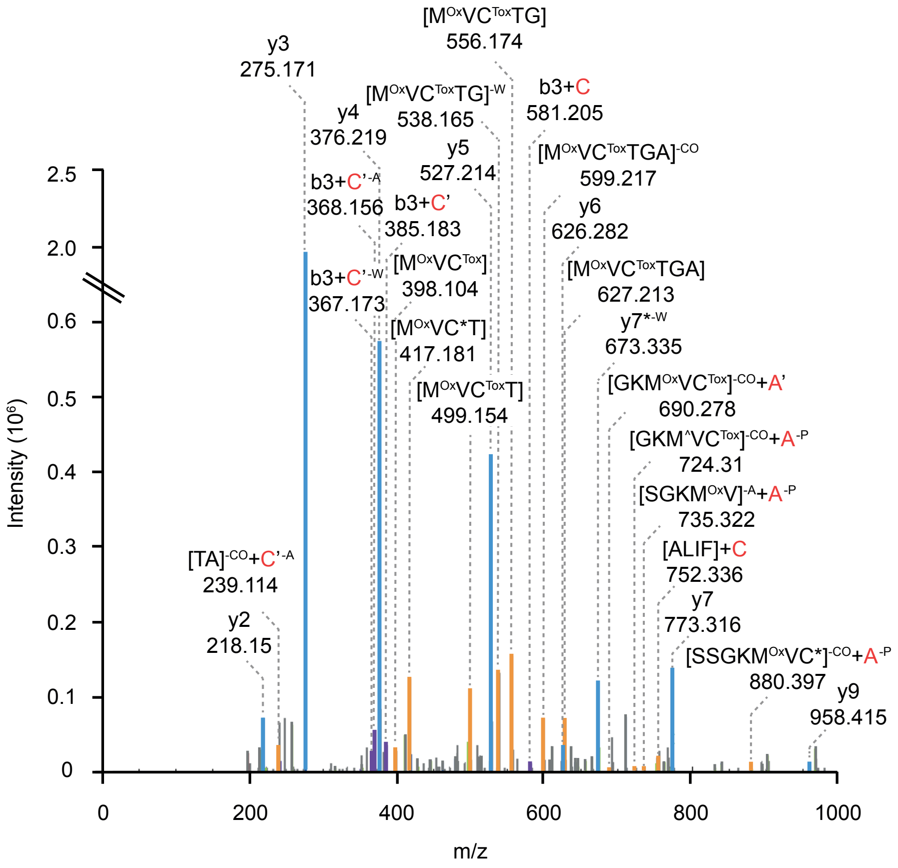

DCP #19

[255]IQNM[Ox]VGSC[Triox]DVK[265] + [TT-HPO_3]

TATA-box-binding protein (P20226)*

m(calculated)			Δm [ppm]	experimental	
peptide	DNA	cross-link		m/z	charge
1256.5377	546.1363	1802.6866	3.4	902.3474	2

* RnpXl identified crosslink as AA-H_2O-HPO_3, however, marker ions and shifted ions clearly point towards TT-HPO_3

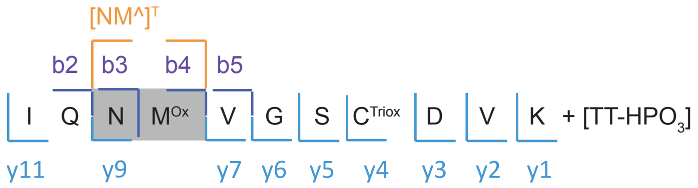

T observed as thymine crosslink

^ neutral loss of CH_4SO

* neutral loss of H_2SO_3 on trioxidized cysteine

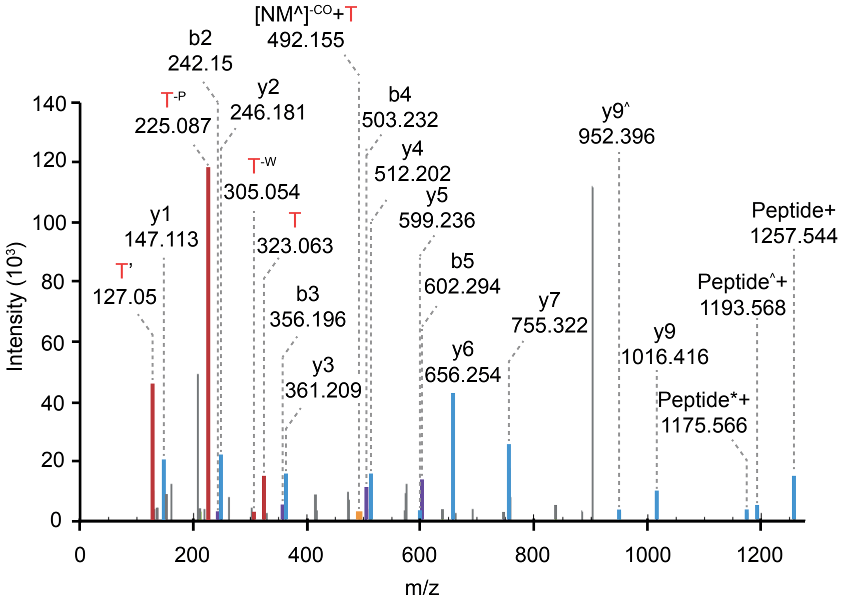

DCP #20

^{178}LDLKTIALR^{186} + [C-HPO$_3$]

TATA-box-binding protein (P20226)

m(calculated)			Δm [ppm]	experimental	
peptide	DNA	cross-link		m/z	charge
1041.6546	227.0906	1268.7452	3.4	635.3821	2

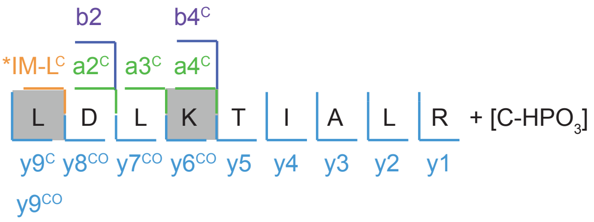

^{C} observed as cytosine crosslink

$^{-/+CO}$ observed with NL/adduct of CO (+27.995)

* a/b ions allow unambiguous localization of this leucine immonium ion

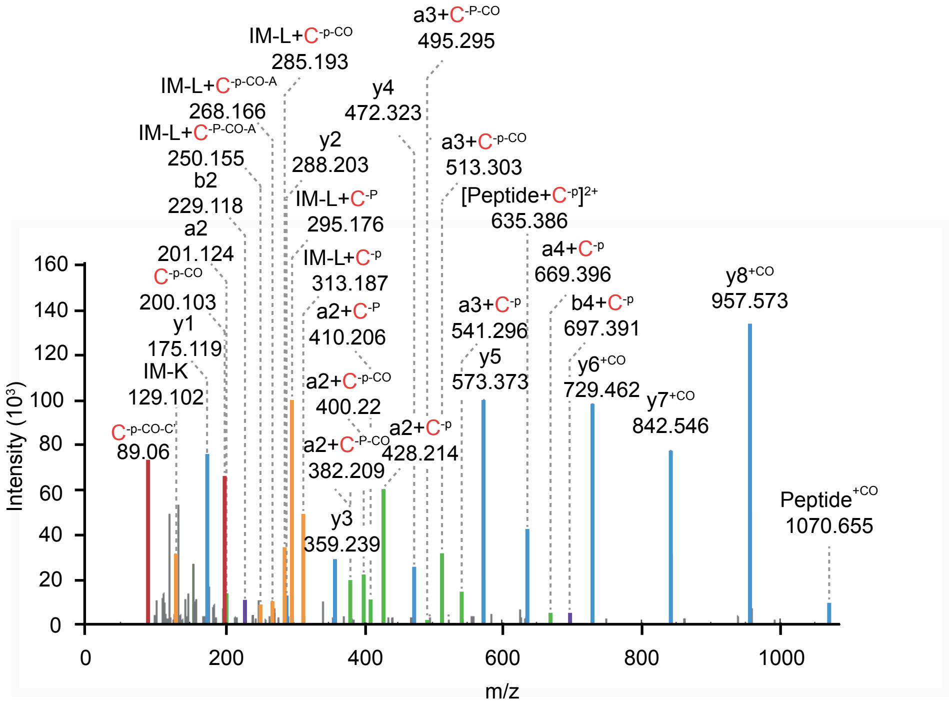

DCP #21

^{584}HLKKAK^{589} + [C-H_2O]

Prospero homeobox protein 1 (P48437)

^{440}HLKKAK^{445} + [C-H_2O]

Prospero homeobox protein 2 (Q8BII1)

m(calculated)			Δm [ppm]	experimental	
peptide	DNA	cross-link		m/z	charge
723.4755	289.0464	1012.5219	3.6	507.2664	2

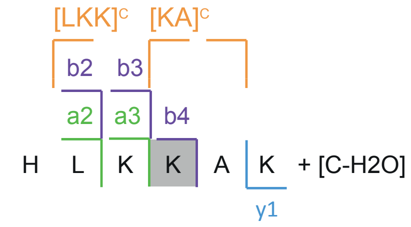

C observed as cytosine crosslink

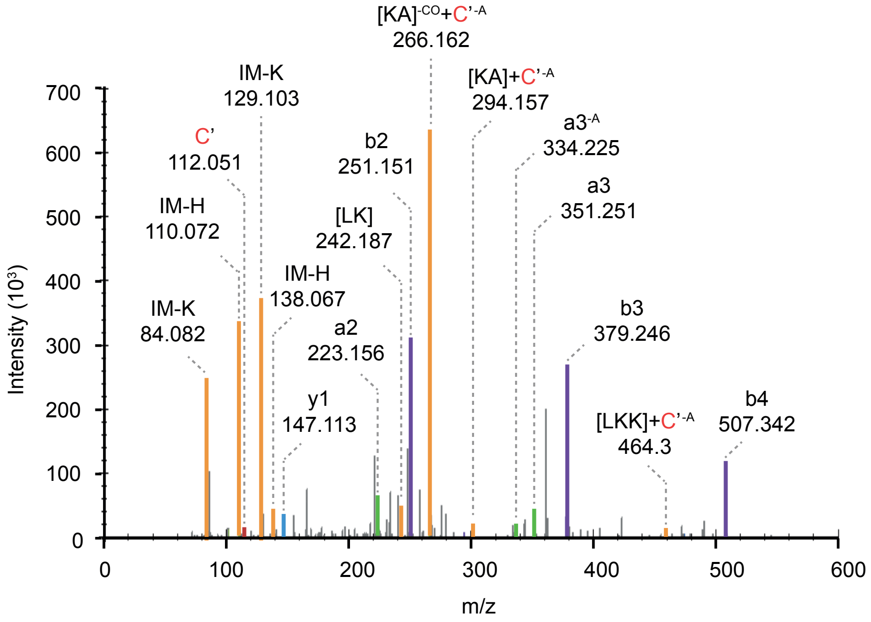

DCP #22

[18]KPLLEK[23] + [TT]

Transcription factor Oct1(Pou2f1) (P25425)* (1,2)

m(calculated)			Δm [ppm]	experimental	
peptide	DNA	cross-link		m/z	charge
726.464	626.1026	1352.5666	10.7	451.8677	3

*1 Proteins Pou2f3, Pou2f2, Hes2 and Mad2l2 not shown

*2 RnpXl identified crosslink as AA-H_2O, however, marker ion and shifted ions clearly point towards TT

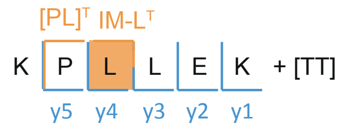

T observed as thymine crosslink

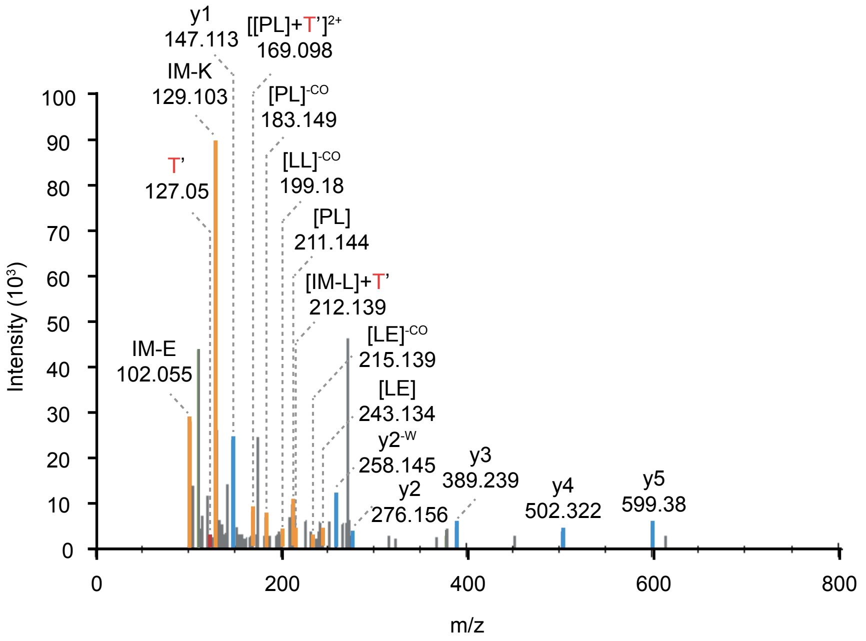

DCP #23

[1325]LQEHVEPVRVK[1335] + [G]

Zinc finger protein 541 (Q0GGX2)

m(calculated)			Δm [ppm]	experimental	
peptide	DNA	cross-link		m/z	charge
1332.7514	347.0631	1679.8145	2.2	560.9467	3

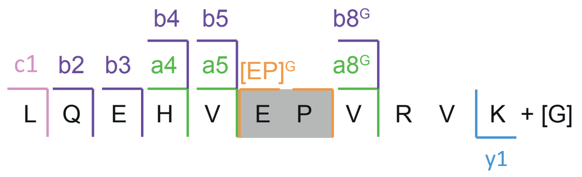

G observed as guanine crosslink

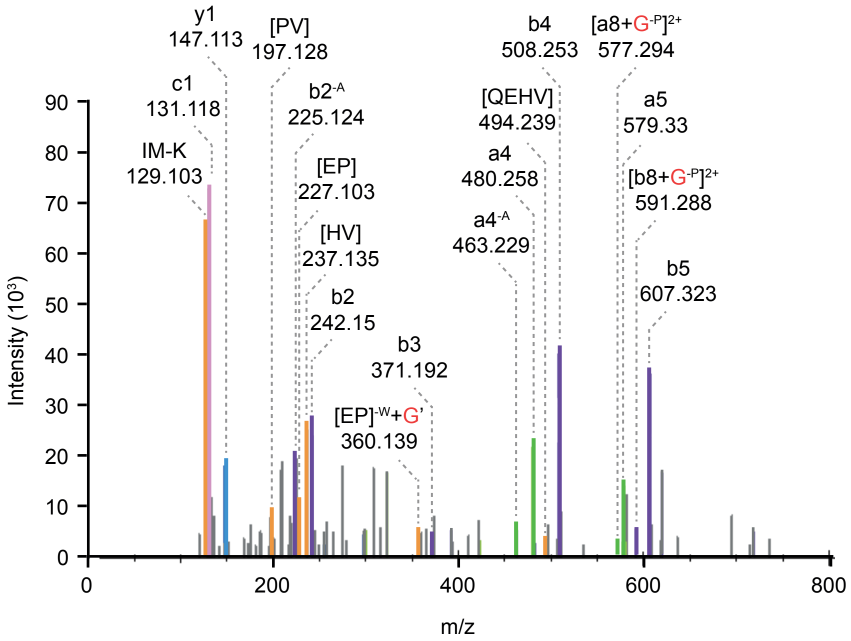

DCP #24

[863]ANEKYGR[869] + [T-HPO_3]

Probable global transcription activator SNF2L1 (Q6PGB8)

m(calculated)			Δm [ppm]	experimental	
peptide	DNA	cross-link		m/z	charge
836.4141	242.0903	1078.5043	0.9	360.5090	3

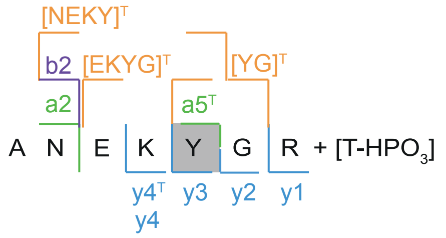

T observed as thymine crosslink

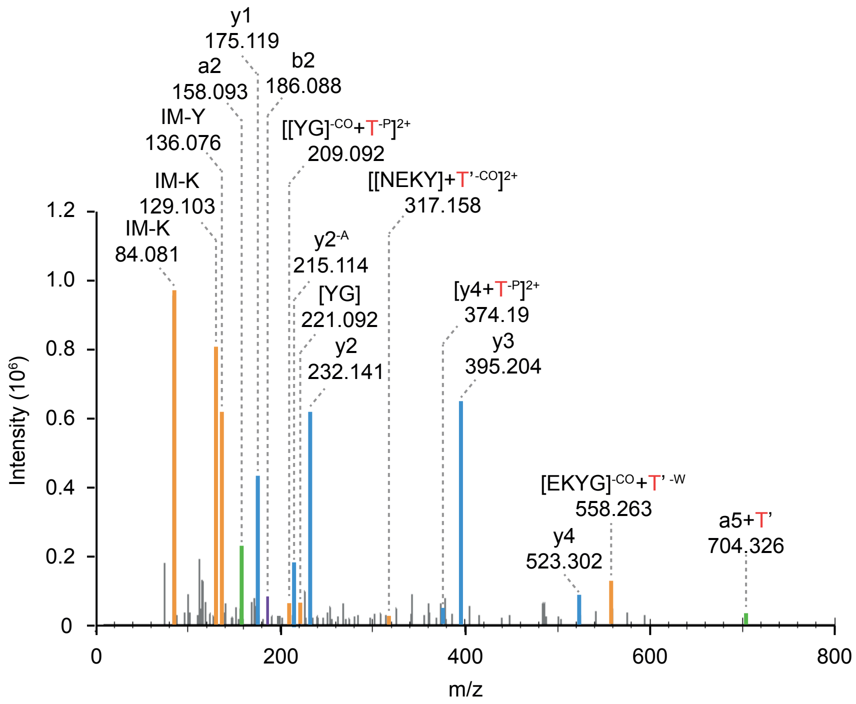

DCP #25

^{282}AFGSSSTLIK^{291} + [G-HPO$_3$]

Zinc finger protein 354C (Q571J5)

m(calculated)			Δm [ppm]	experimental	
peptide	DNA	cross-link		m/z	charge
1009.5444	267.0968	1276.6412	3.7	639.3302	2

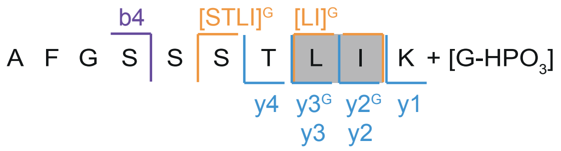

^{G} observed as guanine crosslink

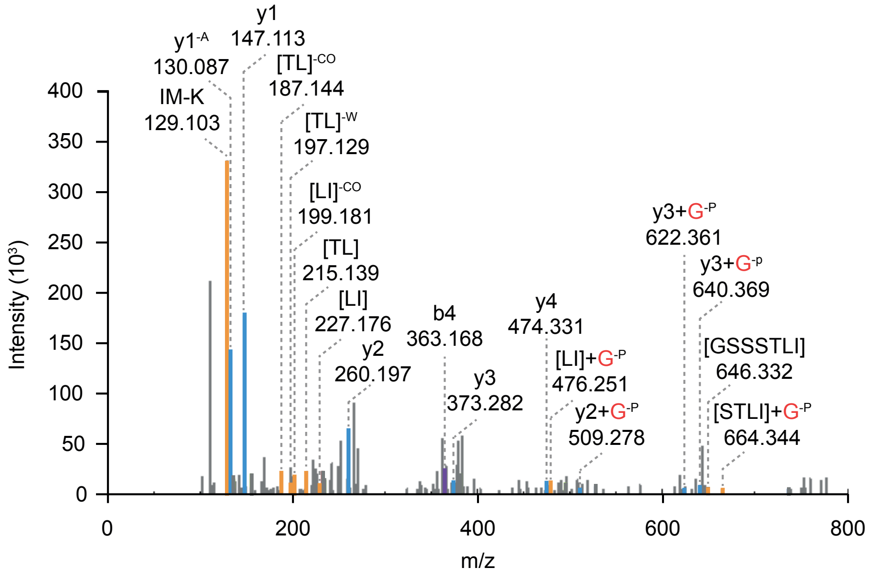

DCP #26

^{444}KDSVVAHKAK^{453} + [AT+HPO_3]

Zinc finger protein 91 (Q62511)

m(calculated)			Δm [ppm]	experimental	
peptide	DNA	cross-link		m/z	charge
1081.6244	715.0805	1796.7049	7.8	599.9136	3

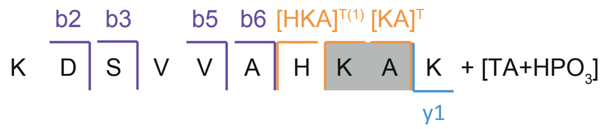

T observed as thymine crosslink

(1) Could also be [AHK]T

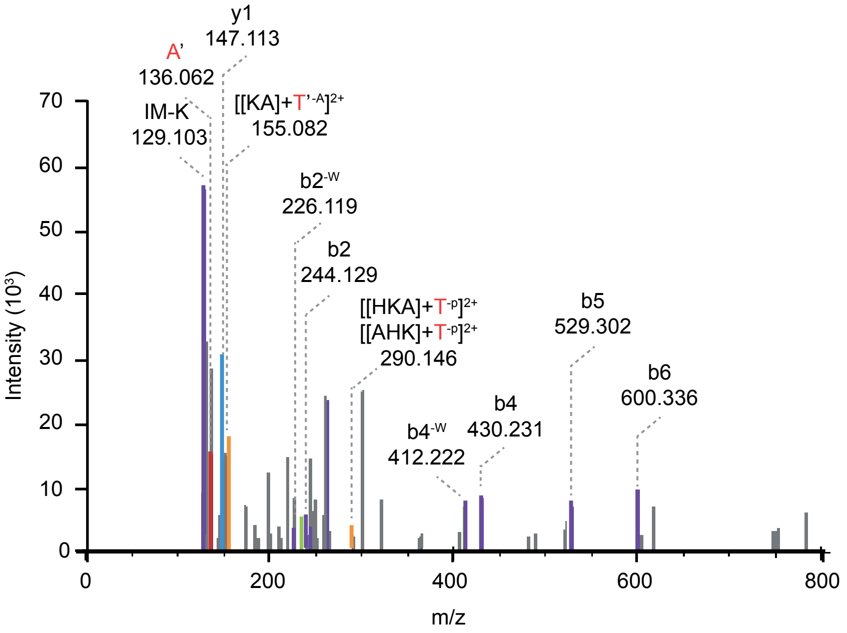

Supplement: Supplementary file 4 — Supplementary Data 1 [file 41467_2020_16837_MOESM4_ESM.pdf]
