# Supplementary material for: Atomic-resolution mapping of transcription factor-DNA interactions by femtosecond laser crosslinking and mass spectrometry
Source: Nat Commun. 2020 Jun 15;11:3019. doi: 10.1038/s41467-020-16837-x (PMC7295792; doi:10.1038/s41467-020-16837-x)
Supplement: Supplementary file 6 — Reporting Summary [file 41467_2020_16837_MOESM6_ESM.pdf]

nature research

Corresponding author(s): Michael Wierer
Christoph Russmann

Last updated by author(s): 5/12/2020

Reporting Summary

Nature Research wishes to improve the reproducibility of the work that we publish. This form provides structure for consistency and transparency in reporting. For further information on Nature Research policies, see Authors & Referees and the Editorial Policy Checklist.

Statistics

For all statistical analyses, confirm that the following items are present in the figure legend, table legend, main text, or Methods section.

n/a	Confirmed	
☒	☐	The exact sample size (*n*) for each experimental group/condition, given as a discrete number and unit of measurement
☒	☐	A statement on whether measurements were taken from distinct samples or whether the same sample was measured repeatedly
☒	☐	The statistical test(s) used AND whether they are one- or two-sided<br>*Only common tests should be described solely by name; describe more complex techniques in the Methods section.*
☒	☐	A description of all covariates tested
☒	☐	A description of any assumptions or corrections, such as tests of normality and adjustment for multiple comparisons
☒	☐	A full description of the statistical parameters including central tendency (e.g. means) or other basic estimates (e.g. regression coefficient) AND variation (e.g. standard deviation) or associated estimates of uncertainty (e.g. confidence intervals)
☒	☐	For null hypothesis testing, the test statistic (e.g. *F*, *t*, *r*) with confidence intervals, effect sizes, degrees of freedom and *P* value noted<br>*Give P values as exact values whenever suitable.*
☒	☐	For Bayesian analysis, information on the choice of priors and Markov chain Monte Carlo settings
☒	☐	For hierarchical and complex designs, identification of the appropriate level for tests and full reporting of outcomes
☒	☐	Estimates of effect sizes (e.g. Cohen's *d*, Pearson's *r*), indicating how they were calculated

Our web collection on statistics for biologists contains articles on many of the points above.

Software and code

Policy information about availability of computer code

Data collection	Therom Q Exactive HF-X Tune Software (v2.9.0.2982), Xcalibur (v4.0.27.19)
Data analysis	Thermo Proteome Discoverer (v2.1.1.21), RNP(xl) plugin (OpenMS nodes v2.0.3), PyMOL (v2.1.1), ImageJ (v1.52a)

For manuscripts utilizing custom algorithms or software that are central to the research but not yet described in published literature, software must be made available to editors/reviewers. We strongly encourage code deposition in a community repository (e.g. GitHub). See the Nature Research guidelines for submitting code & software for further information.

Data

Policy information about availability of data

All manuscripts must include a data availability statement. This statement should provide the following information, where applicable:

- Accession codes, unique identifiers, or web links for publicly available datasets
- A list of figures that have associated raw data
- A description of any restrictions on data availability

The mass spectrometry proteomics data have been deposited to the ProteomeXchange Consortium (http://proteomecentral.proteomexchange.org) via the PRIDE partner repository with the dataset identifier PXD014898.

Field-specific reporting

Please select the one below that is the best fit for your research. If you are not sure, read the appropriate sections before making your selection.

☒ Life sciences ☐ Behavioural & social sciences ☐ Ecological, evolutionary & environmental sciences

Life sciences study design

All studies must disclose on these points even when the disclosure is negative.

Sample size	For each experiment, several samples (n = 2 to 5) of TF-DNA complexes were assembled in-vitro and crosslinked individually. To reach sufficient sample amount for MS-based crosslink detection, samples were pooled prior to TiO2-based enrichment of DNA-modified peptides and measurement by LC-MS/MS.
Data exclusions	No data was excluded from the analyses.
Replication	Due to restrictions in sample amounts and accessibility of the femtosecond laser system, individual experiments were not replicated.
Randomization	Due to the direct comparison of crosslink vs. non-crosslink samples, no randomization was performed.
Blinding	Due to the descriptive nature of the data, no blinding was performed.

Reporting for specific materials, systems and methods

We require information from authors about some types of materials, experimental systems and methods used in many studies. Here, indicate whether each material, system or method listed is relevant to your study. If you are not sure if a list item applies to your research, read the appropriate section before selecting a response.

Materials & experimental systems

n/a	Involved in the study	
☒	☐	Antibodies
☐	☒	Eukaryotic cell lines
☒	☐	Palaeontology
☒	☐	Animals and other organisms
☒	☐	Human research participants
☒	☐	Clinical data

Methods

n/a	Involved in the study	
☒	☐	ChIP-seq
☒	☐	Flow cytometry
☒	☐	MRI-based neuroimaging

Eukaryotic cell lines

Policy information about cell lines

Cell line source(s)	The ES-E14TG2a cell line was received as a gift from Luciano di Croce (CRG, Barcelona)
Authentication	The stem cell character of the cell line was authenticated by MS-based expression analysis of stem cell markers (Nanog, Oct4).
Mycoplasma contamination	The cell line was tested negative for mycoplasma contamination.
Commonly misidentified lines (See ICLAC register)	No misidentified cell line was used in the study.
